# GLP-1 analog liraglutide-induced cardiac dysfunction due to energetic starvation in heart failure with non-diabetic dilated cardiomyopathy

**DOI:** 10.1186/s12933-019-0966-2

**Published:** 2019-11-28

**Authors:** Aya Shiraki, Jun-ichi Oyama, Toshiyuki Nishikido, Koichi Node

**Affiliations:** Department of Cardiovascular Medicine, Faculty of Medicine, Saga University, 5-1-1 Nabeshima, Saga, 849-8501 Japan

**Keywords:** GLP-1, Heart failure, Metabolism

## Abstract

**Background:**

Glucagon-like peptide-1 (GLP-1) reduces cardiovascular events in diabetic patients; however, its counter-protective effects have also been suggested in patients with heart failure and the clear explanation for its mechanisms have not yet been offered.

**Methods:**

The effects of GLP-1 analog on cardiac function and energy metabolism, especially glycemic and lipid metabolisms were elucidated using non-diabetic J2N-k hamsters which showed spontaneous dilated cardiomyopathy. J2N-k hamsters were treated with PBS (HF group), low-dose (HF-L group) or high-dose liraglutide (HF-H group).

**Results:**

In failing heart, GLP-1 analog exerted further deteriorated cardiac function (e.g. positive and negative dP/dt; p = 0.01 and p = 0.002, respectively) with overt fibrosis and cardiac enlargement (heart/body weight, 5.7 ± 0.2 in HF group versus 7.6 ± 0.2 in HF-H group; p = 0.02). The protein expression of cardiac muscles indicated the energy starvation status. Indirect calorimetry showed that failing hearts consumed higher energy and carbohydrate than normal hearts; moreover, this tendency was augmented by GLP-1 analog administration. Upon 10% glucose solution loading with GLP-1 analog administration (HF-H-G group) as complementary experiments, the cardiac function and fibrosis significantly ameliorated, whereas carbohydrate utilization augmented further and lipid utilization reduced more. The prognosis of HF-H-G group also significantly improved (p = 0.025).

**Conclusions:**

Glucagon-like peptide-1 analog caused the relative but desperate shortage of glycemic energy source for the failing cardiac muscles and it may restrict ATP synthesis, resulting in cardiac function deterioration. Therefore, appropriate energy supply and amount of carbohydrate intake should be carefully considered when administrating incretin-related drugs to patients with heart failure.

## Background

Glucagon-like peptide-1 is an incretin hormone, and its analogs and dipeptidyl peptidase-4 (DPP4) inhibitors have been widely used for the treatment of type 2 diabetes mellitus (T2DM) recently. The anti-oxidative and anti-inflammatory effects of the GLP-1 analog liraglutide have been reported in human umbilical vein endothelial cells [1]. Several studies have also reported the cardioprotective effects of GLP-1 and GLP-1-related drugs, including in vitro [2], animal model [2], and clinical studies [3]. In particular, liraglutide [4], semaglutide [5], and albiglutide [6] have been found to reduce the risk of major adverse cardiac events in large-scale clinical trials. GLP-1 analogs have shown anti-atherosclerotic effects possibly by modifying the risk factors, such as glycemic control, insulin resistance, body weight, blood pressure, and lipid profile [7].

However, surprisingly, heart failure (HF) increasingly occurred in patients treated with liraglutide [8] and DPP4 inhibitors in a large-scale clinical trial [9] and some meta-analyses [10, 11]. The mechanisms underlying the role of incretin-related drugs in precipitating HF remain unknown [12].

Therefore, this study aimed to investigate the effects and mechanisms of liraglutide on cardiac dysfunction using hamsters that spontaneously developed HF.

## Methods

### Experimental animals

Male cardiomyopathic (J2N-k; n = 8) and normal (J2N-n; n = 64) hamsters were purchased from Japan SLC Inc., Japan, at the age of 20–27 weeks. J2N-k hamsters were generated from BIO 14.6 hamsters, whose cardiomyopathy are caused by δ-sarcoglycan  mutation and showed exacerbating cardiac function [13]. J2N-k has its origin in BIO 14.6, and thus both of two lineages are innately almost similar; however, CPK levels in J2N-k linage were more rapidly elevated and showed 2 months shorter lifespan as compared with the BIO14.6 linage. The average lifespan of J2N-k is about 37 weeks, and approximately 90% of the cause of death was HF. J2N-k hamsters are recognized as established model of dilated cardiomyopathy (DCM), with mutations also detected in patients with DCM [14]. These hamsters were randomized to receive PBS (HF group) or liraglutide (purchased from Novo nordisk pharma, Denmark). Alzet osmotic pumps (2006) contained about 230 μl of PBS or liraglutide were implanted at the age of 31 weeks. Liraglutide was administered at 20 μg/kg/day (HF-L group) or 100 μg/kg/day (HF-H group) concentrations for 42 days. In the energy-complementary experiments, 10% glucose solution was administrated to hamsters instead of drinking water for 31–37 weeks along with high-dose liraglutide treatment (HF-H-G group).

### Measurement of cardiac functions and tissue sampling

All hamsters (37 weeks old) were anesthetized with 0.75 mg/kg medetomidine, 4 mg/kg midazolam, and 5 mg/kg butorphanol IP. After an echocardiographic evaluation, a catheter was inserted into the left ventricle to measure blood pressure in the LV. After cardiac function measurements, blood samples were collected, HbA1c was measured using Banalyst Ace (USIO INC. Japan), and the rest were stored at − 80 °C. After weighing the hearts and lungs, samples were frozen in liquid nitrogen and stored at − 80 °C or fixed with formalin.

### Western blot analysis

The heart tissue was homogenized in RIPA buffer containing NaF, trypsin inhibitor, leupeptin, glycerophosphate, and orthovanadate. Samples of heart tissue lysate were resolved on SDS-PAGE according to a standard protocol. After being transferred to the membranes, the samples were immunoblotted with primary antibodies, followed by secondary antibodies conjugated to a horseradish peroxidase. Bands were revealed using ECL select western blotting detection reagents (GE Healthcare, Buckinghamshire, UK), and band density was quantified using the Image J software. The following primary antibodies were used: CD36 (#sc-9154) and actin antibodies (#sc-1616) were obtained from Santa Cruz biotechnology (Santa Cruz, CA). GLUT-4 antibody (#2213), cleaved caspase-3 antibody (#9661), phospho-AMPKα (Thr172) antibody (#2535), and LC3A/B (1/2) antibody (#12741), were purchased from Cell Signaling (Danvers, MA).

### ATP measurement

An ATP measurement kit for tissue (TA-100; Toyo B-net, Tokyo, Japan) was used to measure the ATP concentration of heart tissues according to the manufacturer’s protocol. The chemiluminescence by the luciferase reaction was used to measure ATP concentration.

### FFA measurement

Serum samples were obtained without fasting. Serum FFA levels were measured using Free fatty acid fluorometric assay kit (700310, Cayman, MI, USA) according to manufacturer’s instructions.

### Quantification of collagen content in the heart tissue

For conventional light microscopy, hearts were fixed in 4% neutral buffered formaldehyde solution and embedded in paraffin. Sections (8-μm thick) were stained following the Sirius red staining procedure to detect collagens. Red area and whole heart muscle sections were quantified using Image J.

### Respiratory analysis

ARCO-2000 (Arco system, Japan) was used to measure oxygen consumption (VO_2_), carbon dioxide production (VCO_2_), respiratory quotient (RQ), carbohydrate (CHO) and fat (FAT) consumption. It consists of mass spectrometer for respiratory analysis and multi-process monitoring for small animals. After a few days of habituation, data were obtained every 2 min for 2 days. Data were divided into light and dark phases: light phase, 8:00 a.m. to 8:00 p.m., and dark phase, 8 p.m. to 8:00 a.m.

### Statistics

Data comparison in each group was performed with one-way analysis of variance (ANOVA) or Student’s t test with step-down method. Multiple comparisons data were analyzed using the Bonferroni method after ANOVA for all pairwise comparisons. A *p*-value of < 0.05 was deemed statistically significant. All statistical analyses were performed using the SPSS software.

## Results

### Cardiac dysfunction is further exacerbated by administration of GLP-1 analog

**Fig. 1:**
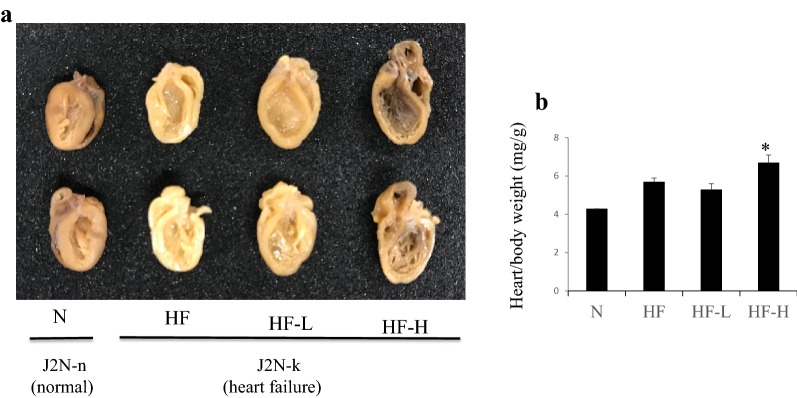
Representative image of hearts from J2N-n and J2N-k hamsters. **a** J2K hamsters, which present dilated cardiomyopathy, were implanted with Alzet 2006 osmotic pumps containing PBS (HF group), low-dose liraglutide (20 µg/kg/day, HF-L group), or high-dose liraglutide (100 µg/kg/day, HF-H group) at 31 weeks of age and sacrificed at 37 weeks of age. The cardiac enlargement at 37 weeks of age is shown for the high-dose liraglutide group. **b** Heart/body weight indicates severity of heart failure. Increased rate of heart/body weight was observed in HF-H group. *p > 0.05 vs. HF group

**Fig. 2:**
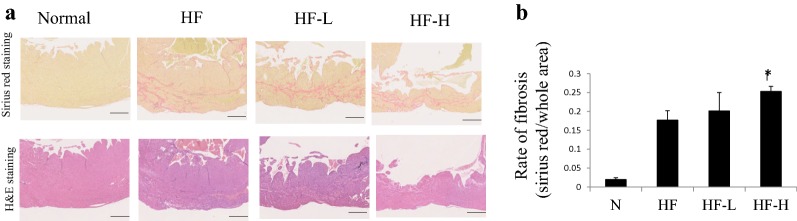
Fibrosis of cardiac muscles. **a** Sirius red staining shows more severe fibrosis in the liraglutide groups among all groups (scale bar: 500 µm). Representative images of anterior wall in short axis of the heart are shown. **b** The rates of fibrosis area/whole area were calculated using the Image J software. Fibrosis was exacerbated by liraglutide administration in a dose-dependent manner. *p < 0.005 (n = 4 in each group) vs. HF group

Table 1 shows the characteristics of healthy hamsters (J2N-n), those (J2N-k) developed genetically dilated cardiomyopathy (DCM) and HF, and those treated with low-dose (HF-L) or high-dose (HF-H) liraglutide. As regards interventions, J2N-k hamsters were treated with low-dose (HF-L group) or high-dose liraglutide (HF-H group). No statistically significant differences in body weight and heart rate among the three groups (HF, HF-L, and HF-H) of J2N-k hamsters; however, the heart/body weight was significantly higher in the HF-H group than those in the HF group. Positive dP/dt (systolic function) and negative dP/dt (diastolic function) were impaired in HF-H groups as compared with the HF group. The LV anterior wall thickness was significantly lower in HF-L and HF-H groups (Table 2). The representative heart samples and heart/body weight are shown in Fig. 1. The high liraglutide group exhibited further cardiac enlargement. Sirius red staining in the HF-H group disclosed significantly more severe fibrosis than that in the HF group (*p* = 0.003; Fig. 2). In Sirius red stain, fibrosis occurred more heavily in anterior-septal wall than in a posterior wall. This may be the one of the reasons for the differences of between anterior wall thickness and posterior wall thickness in this model.

**Table 1:** Biometric and hemodynamic parameters of hamsters with normal heart and heart failure

	Normal hamsters (J2N-n)	PBS treatment (J2N-k)	Low-dose liraglutide treatment (J2N-k)	High-dose liraglutide treatment (J2N-k)
	(n = 8)	(n = 14)	(n = 12)	(n = 13)
Biometry				
Body weight, g	120 ± 4.8	111 ± 2.7	113 ± 2.1	107 ± 2.8
Heart, g	0.51 ± 0.22	0.63 ± 0.20	0.60 ± 0.02	0.72 ± 0.04
Heart/BW, mg/g	4.3 ± 0.0	5.7 ± 0.2	5.3 ± 0.3	6.7 ± 0.4*
Lungs, g	0.75 ± 0.03	0.74 ± 0.04	0.62 ± 0.02	0.85 ± 0.09
Lungs/BW, mg/g	5.8 ± 0.2	6.8 ± 0.4	5.5 ± 0.2	7.9 ± 0.9
Echocardiography				
HR, beats/min	392 ± 18	376 ± 13	412 ± 17	376 ± 19
LVEDD, mm	4.5 ± 0.1	7.5 ± 0.2	7.5 ± 0.2	7.4 ± 0.1
LVESD, mm	2.5 ± 0.1	6.6 ± 0.2	6.4 ± 0.2	6.3 ± 0.2
LV ejection, %	0.81 ± 0.01	0.31 ± 0.01	0.36 ± 0.02	0.35 ± 0.02
LV FS, %	0.45 ± 0.01	0.13 ± 0.01	0.15 ± 0.01	0.15 ± 0.01
LV AW, mm	0.95 ± 0.02	0.81 ± 0.02	0.72 ± 0.02*	0.67 ± 0.01*
LV PW, mm	1.02 ± 0.02	0.84 ± 0.02	0.81 ± 0.02	0.79 ± 0.02
Intracardiac pressure				
Positive dP/dt, mmHg/s	5512 ± 539	3566 ± 235	3100 ± 310	2501 ± 290*
Negative dP/dt, mmHg/s	− 4119 ± 543	− 2669 ± 143	− 2425 ± 281	− 1700 ± 171*
EDP, mmHg	8.65 ± 1.7	8.71 ± 2.4	12.5 ± 4.3	13.8 ± 2.8
Max pressure, mmHg	132 ± 13	80.6 ± 4.2	81.9 ± 14	66.1 ± 5.5
Min pressure, mmHg	4.5 ± 2.0	6.1 ± 2.0	9.3 ± 3.0	9.4 ± 2.0

All values are expressed as mean ± SEM

*HR* heart rate, *LVEDD* left ventricular end-diastolic diameter, *LVESD* left ventricular end-systolic diameter, *FS* fractional shortening, *AW* anterior wall, *PW* posterior wall, *EDP* end-diastolic pressure, *FFA* free fatty acid

Liraglutide treatment groups compared with the PBS treatment group by the step-down method. *P < 0.05

**Table 2:** Biometric and hemodynamic parameters of high-dose liraglutide groups with/without 10% glucose intake

	High-dose liraglutide treatment (J2N-k)	High-dose liraglutide treatment with 10% glucose intake (J2N-k)
	(n = 13)	(n = 12), (n = 5 for LV pressure)
Biometry		
Body weight, g	107 ± 2.8	120.2 ± 2.8^†^
Heart, g	0.72 ± 0.04	0.61 ± 0.03*
Heart/BW, mg/g	6.7 ± 0.4	5.1 ± 0.2^†^
Lung, g	0.85 ± 0.09	0.71 ± 0.03
Lung/BW, mg/g	7.9 ± 0.9	5.9 ± 0.2^†^
Echocardiography		
HR, beats/min	376 ± 19	399 ± 8
LVEDD, mm	7.4 ± 0.1	7.1 ± 0.1
LVESD, mm	6.3 ± 0.2	5.7 ± 0.1^†^
LV ejection, %	0.35 ± 0.02	0.45 ± 0.02^†^
LV FS, %	0.15 ± 0.01	0.20 ± 0.01*
LV AW, mm	0.67 ± 0.01	0.79 ± 0.03^†^
LV PW, mm	0.79 ± 0.02	0.87 ± 0.05^†^
Intracardiac pressure		
Positive dP/dt, mmHg/s	2501 ± 290	4699 ± 981*
Negative dP/dt, mmHg/s	− 1700 ± 171	− 2128 ± 357
EDP, mmHg	13.8 ± 2.8	12.8 ± 2.36
Max pressure, mmHg	66.1 ± 5.5	76.2 ± 6.31
Min pressure, mmHg	9.4 ± 2.0	7.3 ± 0.87

All values were expressed as mean ± SEM. *p > 0.05, ^†^p > 0.005

**Fig. 3:**
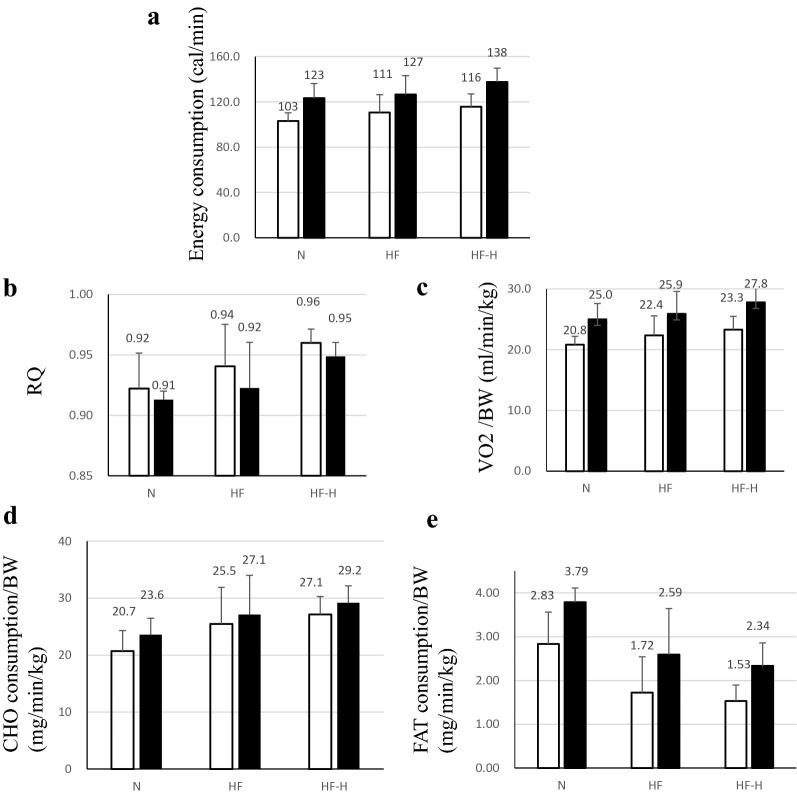
Respiratory gas was analyzed every 2 min in 32-week-old hamsters for 2 days. Average values in light and dark phases are shown in the graph [open bar: light phase, closed bar: dark phase (n = 6 in each group)]. **a** Energy expenditure and **b** RQ tended to be upregulated in the compensated period of heart failure. Liraglutide administration emphasized energy expenditure and RQ upregulation in hamsters with heart failure, indicating increased dependency on carbohydrate as an energy substrate during liraglutide administration. **c** VO_2_/BW, **d** CHO consumption, and **e** FAT consumption in J2N-n hamsters (normal hamsters), J2N-k hamsters with/without administration of high-dose liraglutide. *RQ* respiratory quotient, *VO*_*2*_ oxygen consumption, *CHO* carbohydrate, *FAT* fat

Respiratory gas analysis at aged 32 weeks revealed a relatively high energy consumption and increased CHO and decreased FAT consumption in groups with HF (Fig. 3d, e). Increased CHO reliance was also higher RQ in the HF group (Fig. 3b). These results indicated higher demands for energetic substrate, especially CHO consumption at aged 32 weeks, which were compensated and relatively moderate in HF states and phases in the HF group. When using high-dose liraglutide, this tendency was more noticeable in the HF-H group, marking higher RQ and CHO consumption with lower FAT consumption.

**Fig. 4:**
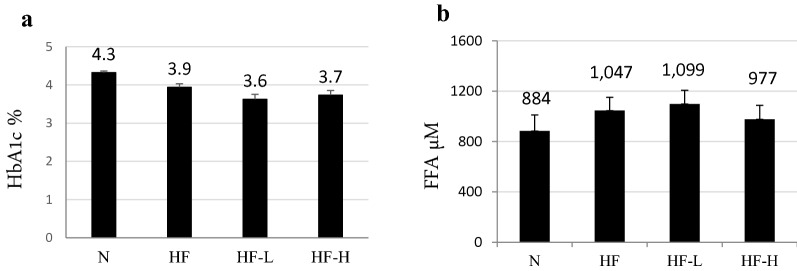
**a** Serum HbA1c and FFA levels in each group. No significant difference was observed among all HF groups, although HbA1c tended to decrease in HF and decrease further in the HF-L and HF-H groups. **b** The serum FFA concentration was more upregulated in the HF group than that in the N group, but it tended to decrease in the HF-H group despite more severe cardiac dysfunction, although it was not statistically significant. *FFA* free fatty acid

Differences in HbA1c and free fatty acid (FFA) concentration were not significant in each group, whereas they tended to increase in the HF group. The tendency of decreased HbA1c levels in HF-L and HF-H is shown in Fig. 4a. In the HF-H group, the FFA level was slightly decreased (Fig. 4b).

**Fig. 5:**
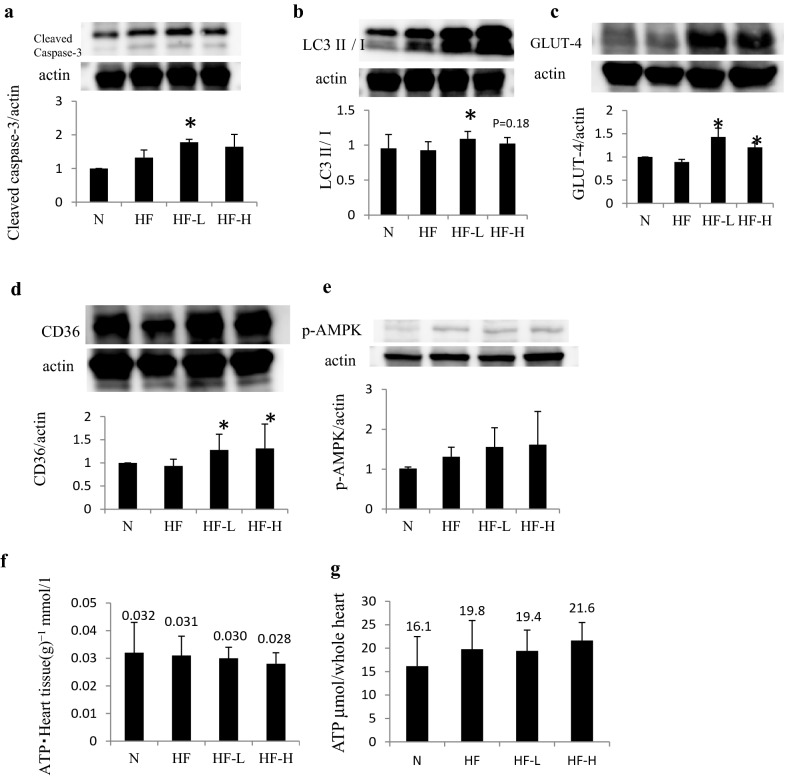
Protein expression and ATP content in the heart muscles of hamsters. **a** Liraglutide administration further upregulated cleaved caspase-3, an apoptosis marker in J2K hamsters. **b** Liraglutide also increased LC3 II/I level that indicated increased autophagy, suggesting that substrate starvation for cardiac muscles occurred in the liraglutide groups. **c**, **d** Liraglutide increased the levels of the glucose transporter GLUT-4 and fatty acid transporter CD36 in cardiac muscles. **e** The upregulation of AMPK phosphorylation tended to occur in the HF groups. **f**, **g** The ATP content in the cardiac tissues slightly decreased in the HF-H groups in 1 g cardiac muscles; however, as a whole heart, higher ATP content was estimated due to the enlarged cardiac tissue in the HF groups. *p < 0.05 vs. HF + PBS group (n = 6 in each group)

Western blotting of heart tissues revealed a tendency of increased cleaved caspase-3 in the HF group and further induction when using liraglutide. An autophagy marker, LC3 II/I, was upregulated in the HF group and even further induced in the HF-L and HF-H groups. In addition, glucose transporter-4 (GLUT-4) and CD36, the substrate glucose and fat transporters, respectively, did not change between the HF and N groups, which particularly increased with GLP-1 analog administration (Fig. 5).

The AMPK phosphorylation tended to be slightly upregulated in HF groups (Fig. 5e). An ATP content in the cardiac muscles is slightly decreased, although no significance was observed in all groups. The ATP content measurements showed a slightly decreased ATP level per gram of heart weight in the HF, HF-L, and HF-H groups (Fig. 5f); however, estimated ATP contents in the whole heart showed slight ATP up-regulation in HF and further increase in the HF-H group, which showed a quite similar tendency in energy consumption measurements in indirect calorimetry in Fig. 3a.

As a result, energy consumption and ATP synthesis were enhanced, which are also exerted in elevated VO_2_ when HF worsened, accompanied with increased CHO and decreased FAT consumption, which showed declined use of lipids as energy source; moreover, GLP-1 analog treatment augmented these phenomena. GLP-1 analog administration has been considered as the cause of remarkable changes in cardiac metabolism, which results in further HF deterioration.

### Additional administration of glucose ameliorated GLP-1-induced cardiac deterioration

A hypothesis that cardiac deterioration was caused by energetic, especially glycemic, deficiency in a failing cardiac muscle due to the GLP-1 agonist has been set. Therefore, whether additional glucose supplementation improved cardiac function in hamsters to provide 10% glucose solution instead of drinking water along with high-dose liraglutide administration (HF-H-G group) is examined.

As a result, hamsters took 6.99 kcal/day from 10% glucose-free intake, and food consumption was decreased. Therefore, the total calorie intake was comparable (24.34 kcal in the HF-H group vs. 24.22 kcal in the HF-H-G group, Table 3).

**Table 3:** Predicted consumption of calories from food and 10% glucose solution intake

Consumption amount		
Food without liraglutide	Food amount (g)/day	6.24 g
	Food calorie (kcal)/day	22.40 kcal
Food with high-dose liraglutide	Food amount (g)/day	6.78 g
	Food calorie (kcal)/day	24.34 kcal
Food without high-dose liraglutide and 10% glucose solution	Food amount (g)/day	4.80 g
	Food calorie (kcal)/day	17.23 kcal
10% glucose solution	Intake volume (ml)/day	17.47 ml
	Intake glucose (g)/day	1.75 g
	Intake calorie (kcal)/day	6.99 kcal
Total		24.22 kcal

Food is a certified diet at 359 kcal/100 g

**Fig. 6:**
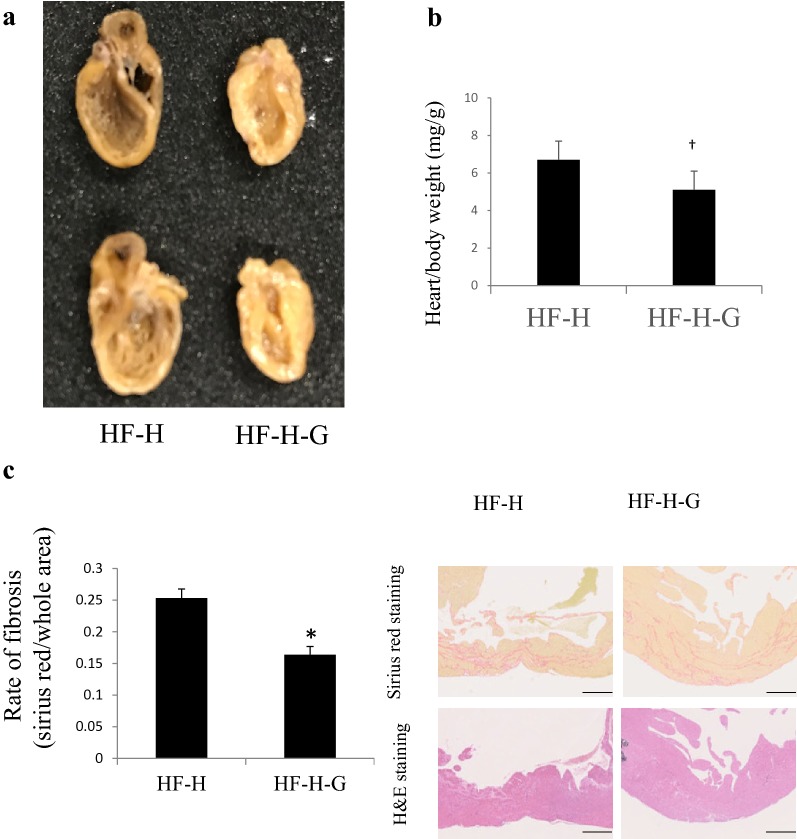
An additional 10% glucose administration to the high-dose liraglutide group ameliorated cardiac dilation and fibrosis. **a** An image representing the cardiac long axis view. **b** Heart/body weight which indicates severity of heart failure, was clearly decreased in HF-H-G group. **c** Sirius red staining showed improved cardiac fibrosis by 10% glucose treatment in combination with high-dose liraglutide (n = 4). Representative images of anterior wall in short axis of the heart are shown (scale bar: 500 µm). *p > 0.05 vs. HF + high-dose liraglutide group. *HF* heart failure, *HF-H-G* heart failure + 10% glucose

**Fig. 7:**
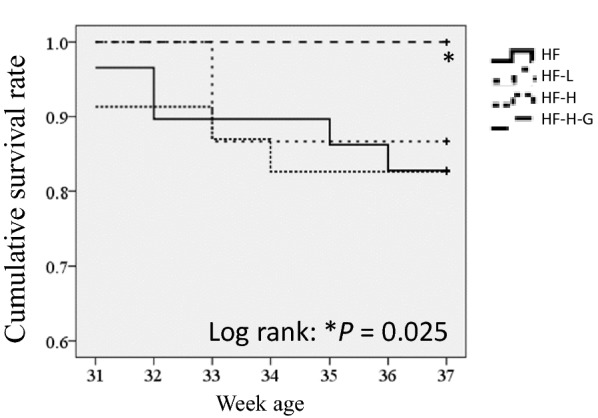
Kaplan–Meier survival curve demonstrates 10% glucose administration attenuated mortality in J2N-K hamsters. The earlier lethality in this group than in HF group was not significant for the liraglutide treatment in this experiment. In contrast, 10% glucose addition saved the mortality of HF hamsters administered with PBS, low-dose, or high-dose liraglutide. Log-rank test, *p = 0.025, HF-H-G vs. HF groups; HF: n = 31; HF + high lira: n = 21; HF + low lira: n = 20; HF + high lira + G: n = 22

**Fig. 8:**
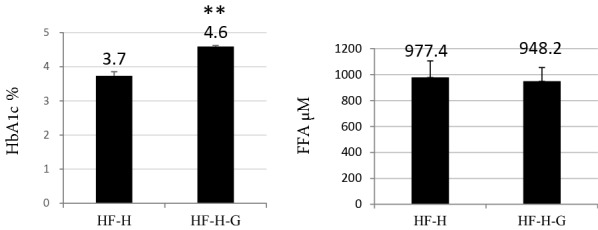
The serum HbA1c and FFA levels in HF-H and HF-H-G groups. The HbA1c level increased by 10% free glucose intake. In contrast, FFA slightly decreased without statistical significance. HF-H: n = 13, HF-H-G: n = 12

Surprisingly, additional administration of glucose completely reversed cardiac enlargement (Fig. 6a), heart/body weight ratio (Fig. 6b 7.6 ± 0.3 in HF-H group vs. 5.1 ± 0.2 in HF-H-G group, *p* < 0.005), lung/body ratio, and LV wall thickness (Table 2) without changing the total calorie intake. In regard to cardiac function, 10% glucose intake ameliorated fractional shortening (0.15% ± 0.01% in the HF-H group vs. 0.20% ± 0.01% in the HF-H-G group, *p *< 0.05) and positive dP/dt (*p* < 0.05). Sirius red staining showed that the rate of fibrosis significantly decreased in the 10% glucose intake group (Fig. 6c). The Kaplan–Meier survival curve showed 10% glucose addition to high-dose liraglutide administration saved the early lethality of J2N-K hamsters (Fig. 7). These effects were accompanied by increased HbA1c levels (3.7% ± 0.1% vs. 4.6% ± 0.0%, *p* < 0.005) and body weight (107 ± 2.8 g vs. 120.2 ± 2.8, *p* < 0.005), whereas FFA concentration was slightly lower without statistical significance than those administered with high-dose liraglutide without 10% glucose intake (HF-H group; Fig. 8). Thus, additional glucose loading prevented cardiac dysfunction amelioration using the GLP-1 analog.

**Fig. 9:**
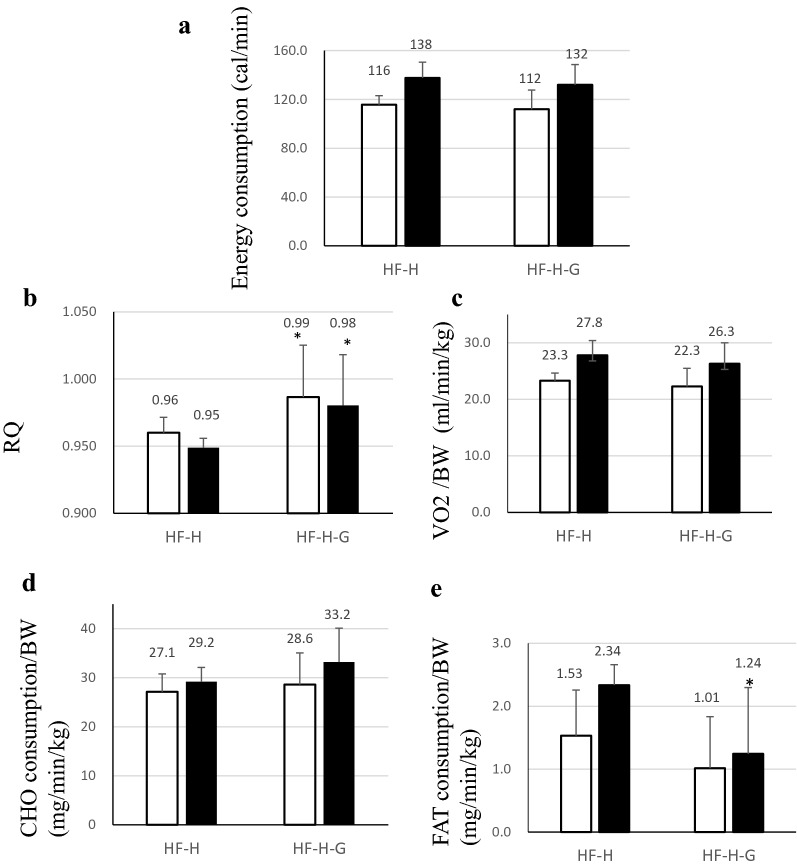
Respiratory gas was captured every 2 min in 32-week-old hamsters for 2 days. Average values of light and dark phases are shown in the graph [open bar: light phase, closed bar: dark phase (n = 6 in each group)]. **a** Energy expenditure was similar for administration of high-dose liraglutide with/without 10% glucose; however, **b** RQ was significantly upregulated in the group with 10% glucose administration. **c** VO_2_/BW in hamsters with heart failure treated with liraglutide with/without 10% glucose. Liraglutide administration emphasized RQ upregulation in hamsters with heart failure, indicating increased dependency on (**d**) carbohydrate as an energy substrate during liraglutide administration and thereby suggesting that glucose was efficiently metabolized as an energy source. **e** In contrast, fat oxidation reduced with addition of 10% glucose. *RQ* respiratory quotient, *VO*_*2*_ oxygen consumption, *CHO* carbohydrate, *FAT* fat. *p > 0.05 vs. HF-H group

Moreover, indirect calorimetry showed that the highest RQ (0.99 in light phase and 0.98 in dark phase) and CHO and lowest FAT consumption were considered reasonable for consistent total energy consumption in the HF-H group as observed in the HF-H-G group (Fig. 9).

**Fig. 10:**
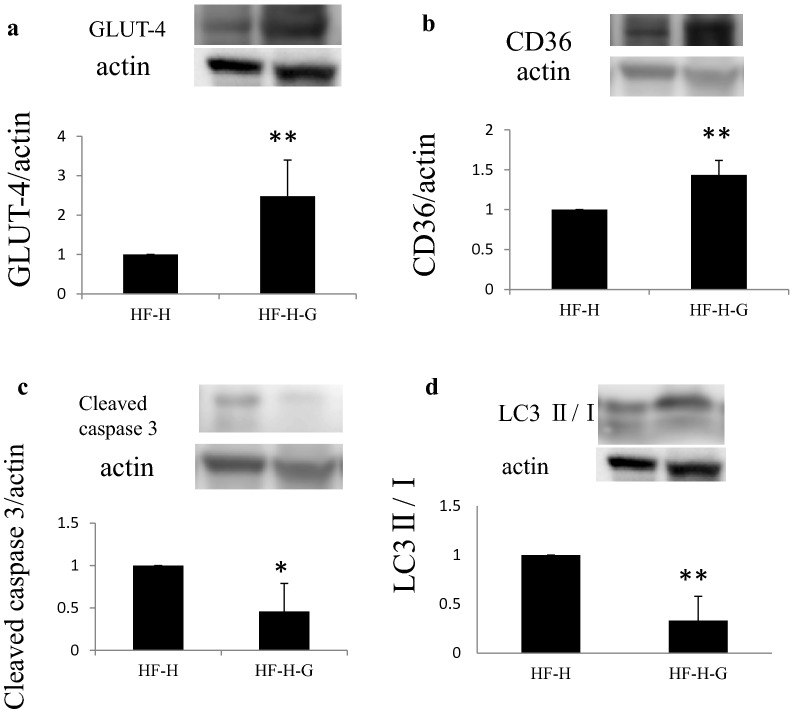
Changed protein expression in cardiac muscles by 10% glucose administration for heart failure deteriorated by high-dose liraglutide. **a**, **b** GLUT-4 and CD36 were upregulated further. **c**, **d**  The levels of the apoptotic marker cleaved caspase-3 and autophagy marker LC3 II/I significantly reduced with 10% glucose administration. n = 6 in each group

Western blotting analysis revealed that cleaved caspase-3 and LC3 II/I levels were significantly lower in the HF-H-G group than those in the HF-H group. In contrast, GLUT-4 and CD36 expression levels in the HF-H-G group were further augmented than those in the high-dose liraglutide group without glucose (Fig. 10).

Based on these results, GLP-1 analog compulsorily augmented the glucose demand in failing hearts that already depend more on glucose rather than fatty acids. Therefore, cardiac function could be maintained when adequate glucose supply could meet the excessive glucose demand caused by liraglutide administration in failing hearts, which had been already over-demanding for carbohydrates.

## Discussion

The major findings in this study were as follows: (1) GLP-1 analog exacerbated cardiac dysfunction and (2) adequate glucose loading as energy substrate prevented HF deterioration.

The energy starvation hypothesis as a cause of HF has been drawing renewed attention recently [15, 16]. Our study suggested that, in line with “An Engine Out of Fuel” hypothesis [16], the cardiac muscle treated with liraglutide showed energy-starved expression, increasing the LC3 II/I ratio that resulted in autophagy occurrence, recycling system against energy depletion.

Previous studies revealed that failing hearts reduced FFA uptake and oxidation and, in contrast, elevated the glucose uptake in advanced HF [17–19], which were supported in our study, as observed by the increased CHO/FAT consumption rate in dilated cardiomyopathy. Although the mechanism remains uncertain in failing hearts, metabolic preference of substrates has been generally considered to change from lipid utilization to carbohydrate utilization [16, 20], although several researches elucidated malnutrition expressing that lower levels of low-density lipoprotein (LDL) cholesterol were correlated with worse HF prognosis. Clinical researches clearly showed that the lower the cholesterol level, the worse the prognosis in patients with HF [21]. Although lipid-lowering therapy with HMG-CoA reductase is recommended for patients with acute myocardial infarction, poor nutrition has a worse outcome in patients with HF, and low LDL levels were associated with worse outcomes than those in high LDL levels, known as the “lipid paradox.” Impaired myocardial energy production is associated with decreased ATP and phosphocreatine concentrations. This depletion appears to result from mitochondrial oxidation compromise in both fatty acids and carbohydrates, accompanied with a compensatory increase in glucose uptake and glycolysis [20]. However, effects and metabolism of using anti-diabetic drugs on failing heart have not been sufficiently concluded in previous studies. In our study, the GLP-1 analog was found to augment the features of energetic states in HF, resulting in the upregulation of carbohydrate consumption and reduced lipid consumption.

Although liraglutide treatment compromised failing heart, it still exerts protective effects when adequate amount of carbohydrate was supplied to the hearts. High CHO consumption and high RQ were 0.99 in the light phase and 0.98 in the dark phase in liraglutide with adequate glucose group, resulting in the highest cardiac function performance as compared with all HF groups, including the PBS group. Increased glucose/fat rate but not total calories in energy substrate saved the failing heart from starvation, prevented fibrosis, cardiac muscle thinning, and heart enlargement. In regard to protein expression, GLUT-4, the main glucose transporter in cardiac muscles, was upregulated in this study. This result was consistent with other studies demonstrating that GLUT-4 was induced by DPP4 inhibitors and liraglutide in the cardiac tissues, effectively allowing glucose into the tissues [22, 23]. Our result is also compatible with reports from Ramírez et al. [24] that sitagliptin increased glucose assimilation in detriment of fatty acid.

Originally, GLP-1 is secreted from the intestines after a meal ingestion and responds to the high glucose concentration in the blood and ends up letting glucose into the muscles and liver [25]. GLP-1 analog augments metabolic reliance on glucose in cardiac muscles and other organs, increasing insulin concentration and GLUT-4 expression in tissues and insulin secretion from the pancreas [26]. Interestingly, Hausenloy et al. [27] reported that the cardioprotective effect of GLP-1 for myocardial infarction was glucose-dependent, and GLP-1 and sitagliptin, a DPP4 inhibitor, did not reduce the infarct size with abnormal glucose level of 5 mmol/l, but high glucose level of 11 mmol/l in ex vivo and > 7–8 mmol/l in vivo study. Our results are in consistent with their findings that the cardioprotective effect of GLP-1 may occur only when the glucose concentration level is high. Kyhl et al. also reported that liraglutide treatment lacked any favorable alterations after acute MI in non-diabetic rats [28], this result might be caused by expected relatively low to normal level of glucose concentration.

In this study, dilated and weakened cardiac muscles exerted increased glucose dependency more than healthy hearts; moreover, glycemic reliance is much more reinforced by GLP-1 analog in a failing heart. Liraglutide-treated failing heart was considered to have glucose shortage as a fuel because of the fact that adequate glucose supply dramatically improved cardiac function and their early mortality. As HbA1c levels between high-dose liraglutide and PBS groups were not significant, no apparent hypoglycemia has occurred. However, there may have been a relative shortage in failing hearts due to the shift in energy utilization from lipids to carbohydrates. In fact, liraglutide is clinically used against obesity for non-diabetic patients [29–31], and no severe hypoglycemia has been reported. Therefore, GLP-1 agonist is recognized to reduce the glucose levels only when in hyperglycemia such as immediately after the meal. When the blood glucose level is low, GLP-1 cannot stimulate insulin secretion and inhibit glucagon secretion to maintain normoglycemia [32]. Therefore, our findings do not support the idea that apparent hypoglycemia caused HF exacerbation. Failing cardiac muscle demand more glucose; furthermore, liraglutide may boost this glucose dependency in terms of RQ upregulation and CHO consumption in the high-dose liraglutide group.

In this study, the influence of food and drink intake quantity according to liraglutide use cannot be excluded. GLP-1 reportedly inhibits gastric motility and emptying and suppresses appetite with body weight and fat tissue loss [33]. However, energy intake was almost equal in all groups, and energy consumption was relatively higher in the liraglutide-treated group as compared with the HF group. Therefore, shortage of total energy intake was not the mechanism to explain the HF worsening in this experiment.

A concern on another important substrate for heart muscles and lipid regulation has been reported. Although CD36, a lipid transporter, was significantly upregulated in cardiac tissues treated with liraglutide that relatively reduced serum FFA levels, FAT consumption in gas analysis was reduced, suggesting that fat consumption was decreased in reality. In fact, when glucose was additionally administered, cardiac dysfunction improved. This implies that liraglutide induced GLUT-4 in cardiac muscles, and glucose was effectively and preferably used for cardiac fertilization.

Increase serum FFA is thought to correlate with HF severity caused by the hyperadrenergic state [16]; however, the relatively low FFA level was observed in failing hearts treated with high-dose liraglutide. GLP-1 is reported to act on enterocyte, the cell responsible for lipid absorption and chylomicron assembly and secretion. Activation of GLP-1R signaling including liraglutide rapidly lowers plasma concentration of ApoB, chylomicrons, and triglycerides in vivo [33, 34]. Therefore, low FFA levels may be caused by the inhibitive effects of liraglutide on lipid absorption and released into the blood. Hamsters treated with high-dose liraglutide might not be able to use fatty substrate due to suppressed absorption and smaller amount of fat intake in the HF-H-G group. This lipid substrate depletion in the body might be one of the reasons of increased glucose feasibility in the high liraglutide groups.

Another explanation can be made based on the Randle cycle hypothesis, known as the “glucose-fatty acid cycle” to describe the association between fuel flux and fuel selection in tissues. Originally, lipid utilization inhibits glucose metabolism in tissues and has been known to play a role in insulin resistance; therefore, glucose utilization has also been reported to inhibit lipid metabolism in muscles and vice versa, thereby increasing malonyl-CoA that signals glucose utilization and controls long-chain fatty acid entry and oxidation in the mitochondria [35]. In this article, we focused metabolism of heart muscles, however, liraglutide has pleiotropic effects in many organs such as digestive system, skeletal muscle and brain. We have to consider those extracardiac effects, especially liver and skeletal muscle, which have strong relations regarding glucose flow in the body. Reduced glucose production in liver and increased glucose uptake in skeletal muscle may cause the limitation of glucose use in the heart.

It is reported that liraglutide increases 6–10 bpm heart rate by stimulation of the sinus node, and it could be through sympathetic nervous system in patients [36]. In our experiments, however, we could not reproduce heart rate upregulation, it may be possibly caused by anesthesia which affects heart rate when we performed cardiac catheterization and echocardiogram to obtain data including heart rate.

We showed exacerbated fibrosis in liraglutide administration along with HF, contradicted reports also exist. Zhang et al. [37] demonstrated that liraglutide treatment attenuated angiotensin II-induced tissue fibrosis, Zhao [38] found liraglutide alleviates cardiac fibrosis in STZ-induced diabetic cardiomyopathy. These conflictions might arise from the differences in animal model; pressure loaded hypertensive heart disease, high glucose and high fat induced cardiomyopathy and/or HF with reduced ejection fraction. Aoyama et al. also noted that DPP4 inhibition alleviates shortage of GLP-1 induced by thoracic aortic constriction (TAC) and reported its protective effect. However, TAC model is pressure loaded model with induced hypertrophy of the heart, difference of etiology compared to present study may result in different conclusion [39].

Certainly, diabetic patients have been known to be highly at risk of developing heart failure [40]. Most clinical articles reported that DPP-4 inhibitors and GLP-1 analogs are neutral for adverse effects on HF; however, several remarkable studies have already been reported, and results of recent large-scale clinical trials are attracting public attention. The SAVOR-TIMI53 trial resulted in increased hospitalization for HF due to saxagliptin use (hazard ratio [HR]: 1.27, 95% confidence interval [CI] 1.07–1.51). Although the TECOS study was completely neutral (HR: 1.00, 95% CI 0.83–1.20), DPP 4 inhibitor in the EXAMINE trial tended to increase the hospitalization for HF (HR: 1.19: 95% CI 0.90–1.58). Moreover, a population-based retrospective cohort study described that sitagliptin use was not associated with increased risk of hospitalization or death, but was associated with increased risk of HF-related hospitalization in T2DM patients with pre-existing HF [41].

GLP-1 receptor antagonists have been expected beneficial effects extend to the cardiovascular system [42, 43]. However, in the FIGHT trial, a randomized clinical trial that investigated whether liraglutide improves the prognosis in patients with advanced HF, the primary end-point was that liraglutide could not improve the mortality [8]. However, especially regarding death or rehospitalization for HF, GLP-1 analog tended to worsen (HR: 1.30: 95% CI 0.92–1.83), although no statistically significant difference was observed. No favorable effects were observed for advanced HF with reduced LVEF. Moreover, it might be some differences between DPP4 inhibitor and GLP-1 agonist in HF patients. In another clinical cohort study, which figured that the use of GLP-1 agonists was associated with an increased risk of HF hospitalization compared to DPP4 inhibitors [44].

In experimental studies, saxagliptin impairs cardiac contraction by inhibiting Ca^2+^/calmodulin-dependent protein kinase II-phospholamban-sarcoplasmic reticulum Ca^2+^-ATPase 2a axis and Na^+^–Ca^2+^ exchanger function in Ca^2+^ extrusion [45]. Genetic DPP-4 depletion exhibits impaired cardiac function and accelerated cardiac fibrosis in TAC-operated high fat-fed mice [46]. Exendin-4, a GLP-1 receptor agonist, can increase the heart rate and blood pressure [47]. These findings may be complicit in malice.

Therefore, many studies including clinical trials are still investigating the possibility and mechanisms whether incretin-related drugs may deteriorate cardiac function; however, the truthful and precise mechanism remains to be clarified, and their potential association with serious adverse effects including HF still exists. In this study, carbohydrates and lipids were investigated as energy sources for the heart and showed that GLP-1 analog deteriorates cardiac function in failing hearts. Furthermore, the deterioration of HF has been clarified to be prevented by adequate glucose supplementation with liraglutide administration. Failing heart may shift from lipid to carbohydrate utilization, and incretin-related drugs may result in insufficient carbohydrate supply to the heart and fail to exert protective effects to the cardiac function.

## Conclusions

In HF situations, energy metabolism in the heart has shifted from lipid to carbohydrate, and the administration of GLP-1 agonist causes further cardiac dysfunction. Furthermore, adequate glucose supplementation improved HF. The mechanisms are suggested as follows: (1) energy starvation occurred in the cardiac muscle by liraglutide administration, (2) cardiac function was improved because sufficient supplementation of carbohydrate satisfied increased energetic, especially glycemic demand from the failing heart treated with GLP-1 agonist.

The mechanism behind the cardiac function deterioration when treated with GLP-1 analog is considered to be caused by imbalanced carbohydrate demand and supplementation in the heart. This study has limitations because these are animal model experiments, further clinical studies should be conducted. However, the results in this study elucidate the adverse effects of GLP-1 analog on failing heart and the importance of glycemic metabolisms as an energy source, especially when using anti-metabolic drugs. Therefore, the glycemic control in patients with HF need to be different from those in patients without HF.

## Data Availability

The used and/or analyzed datasets are available from the corresponding author on reasonable request.

## Abbreviations

GLP-1: glucagon-like peptide-1
DPP4: dipeptidyl peptidase-4
HF: heart failure
VO_2_: oxygen consumption
VCO_2_: carbon dioxide production
CHO: carbohydrate
FAT: fat
FFA: free fatty acid
GLUT-4: glucose transporter-4

## References

[1] Shiraki A, Oyama J, Komoda H, Asaka M, Komatsu A, Sakuma M, Kodama K, Sakamoto Y, Kotooka N, Hirase T, Node K (2012). The glucagon-like peptide 1 analog liraglutide reduces TNF-α-induced oxidative stress and inflammation in endothelial cells. Atherosclerosis.

[2] Ravassa S, Zudaire A, Díez J (2012). GLP-1 and cardioprotection: from bench to bedside. Cardiovasc Res.

[3] Sokos GG, Nikolaidis LA, Mankad S, Elahi D, Shannon RP (2006). Glucagon-like peptide-1 infusion improves left ventricular ejection fraction and functional status in patients with chronic heart failure. J Card Fail.

[4] Marso SP, Daniels GH, Brown-Frandsen K, Kristensen P, Mann JF, Nauck MA, Nissen SE, Pocock S, Poulter NR, Ravn LS, Steinberg WM, Stockner M, Zinman B, Bergenstal RM, Buse JB, LEADER Steering Committee, LEADER Trial Investigators (2016). Liraglutide and cardiovascular outcomes in type 2 diabetes. N Engl J Med.

[5] Marso SP, Bain SC, Consoli A, Eliaschewitz FG, Jódar E, Leiter LA, Lingvay I, Rosenstock J, Seufert J, Warren ML, Woo V, Hansen O, Holst AG, Pettersson J, Vilsbøll T, SUSTAIN-6 Investigators (2016). Semaglutide and cardiovascular outcomes in patients with type 2 diabetes. N Engl J Med.

[6] Hernandez AF, Green JB, Janmohamed S, D’Agostino RB, Granger CB, Jones NP, Leiter LA, Rosenberg AE, Sigmon KN, Somerville MC, Thorpe KM, McMurray JJV, Del Prato S, Harmony Outcomes committees and investigators (2018). Albiglutide and cardiovascular outcomes in patients with type 2 diabetes and cardiovascular disease (Harmony Outcomes): a double-blind, randomised placebo-controlled trial. Lancet.

[7] Andrikou E, Tsioufis C, Andrikou I, Leontsinis I, Tousoulis D, Papanas N (2018). GLP-1 receptor agonists and cardiovascular outcome trials: an update. Hellenic J Cardiol.

[8] Margulies KB, Hernandez AF, Redfield MM, Givertz MM, Oliveira GH, Cole R, Mann DL, Whellan DJ, Kiernan MS, Felker GM, McNulty SE, Anstrom KJ, Shah MR, Braunwald E, Cappola TP, NHLBI Heart Failure Clinical Research Network (2016). Effects of liraglutide on clinical stability among patients with advanced heart failure and reduced ejection fraction: a randomized clinical trial. JAMA.

[9] Scirica BM, Braunwald E, Raz I, Cavender MA, Morrow DA, Jarolim P, Udell JA, Mosenzon O, Im K, Umez-Eronini AA, Pollack PS, Hirshberg B, Frederich R, Lewis BS, McGuire DK, Davidson J, Steg PG, Bhatt DL, SAVOR-TIMI 53 Steering Committee and Investigators (2014). Heart failure, saxagliptin, and diabetes mellitus: observations from the SAVOR-TIMI 53 randomized trial. Circulation.

[10] Monami M, Dicembrini I, Mannucci E (2014). Dipeptidyl peptidase-4 inhibitors and heart failure: a meta-analysis of randomized clinical trials. Nutr Metab Cardiovasc Dis.

[11] Clifton P (2014). Do dipeptidyl peptidase IV (DPP-IV) inhibitors cause heart failure?. Clin Ther.

[12] Zannad F, Rossignol P (2019). Dipeptidyl peptidase-4 inhibitors and the risk of heart failure. Circulation.

[13] Sakamoto A, Ono K, Abe M, Jasmin G, Eki T, Murakami Y, Masaki T, Toyo-oka T, Hanaoka F (1997). Both hypertrophic and dilated cardiomyopathies are caused by mutation of the same gene, delta-sarcoglycan, in hamster: an animal model of disrupted dystrophin-associated glycoprotein complex. Proc Natl Acad Sci USA.

[14] Luk A, Ahn E, Soor GS, Butany J (2009). Dilated cardiomyopathy: a review. J Clin Pathol.

[15] Ingwall JS, Weiss RG (2004). Is the failing heart energy starved? On using chemical energy to support cardiac function. Circ Res.

[16] Heusch G, Libby P, Gersh B, Yellon D, Böhm M, Lopaschuk G, Opie L (2014). Cardiovascular remodelling in coronary artery disease and heart failure. Lancet.

[17] Osorio JC, Stanley WC, Linke A, Castellari M, Diep QN, Panchal AR, Hintze TH, Lopaschuk GD, Recchia FA (2002). Impaired myocardial fatty acid oxidation and reduced protein expression of retinoid X receptor-alpha in pacing-induced heart failure. Circulation.

[18] Dávila-Román VG, Vedala G, Herrero P, de las Fuentes L, Rogers JG, Kelly DP, Gropler RJ (2002). Altered myocardial fatty acid and glucose metabolism in idiopathic dilated cardiomyopathy. J Am Coll Cardiol.

[19] Neglia D, De Caterina A, Marraccini P, Natali A, Ciardetti M, Vecoli C, Gastaldelli A, Ciociaro D, Pellegrini P, Testa R, Menichetti L, L’Abbate A, Stanley WC, Recchia FA (2007). Impaired myocardial metabolic reserve and substrate selection flexibility during stress in patients with idiopathic dilated cardiomyopathy. Am J Physiol Heart Circ Physiol.

[20] Lopaschuk GD (2017). Metabolic modulators in heart disease: past, present, and future. Can J Cardiol.

[21] Lu YW, Lu SF, Chou RH, Wu PS, Ku YC, Kuo CS, Chang CC, Tsai YL, Wu CH, Huang PH (2018). Lipid paradox in patients with acute myocardial infarction: potential impact of malnutrition. Clin Nutr.

[22] Giannocco G, Oliveira KC, Crajoinas RO, Venturini G, Salles TA, Fonseca-Alaniz MH, Maciel RM, Girardi AC (2013). Dipeptidyl peptidase IV inhibition upregulates GLUT4 translocation and expression in heart and skeletal muscle of spontaneously hypertensive rats. Eur J Pharmacol.

[23] Noyan-Ashraf MH, Shikatani EA, Schuiki I, Mukovozov I, Wu J, Li RK, Volchuk A, Robinson LA, Billia F, Drucker DJ, Husain M (2013). A glucagon-like peptide-1 analog reverses the molecular pathology and cardiac dysfunction of a mouse model of obesity. Circulation.

[24] Ramírez E, Picatoste B, González-Bris A, Oteo M, Cruz F, Caro-Vadillo A, Egido J, Tuñón J, Morcillo MA, Lorenzo Ó (2018). Sitagliptin improved glucose assimilation in detriment of fatty-acid utilization in experimental type-II diabetes: role of GLP-1 isoforms in Glut4 receptor trafficking. Cardiovasc Diabetol.

[25] Vyas AK, Yang KC, Woo D, Tzekov A, Kovacs A, Jay PY, Hruz PW (2011). Exenatide improves glucose homeostasis and prolongs survival in a murine model of dilated cardiomyopathy. PLoS ONE.

[26] Li Z, Ni CL, Yao Z, Chen LM, Niu WY (2014). Liraglutide enhances glucose transporter 4 translocation via regulation of AMP-activated protein kinase signaling pathways in mouse skeletal muscle cells. Metabolism.

[27] Hausenloy DJ, Whittington HJ, Wynne AM, Begum SS, Theodorou L, Riksen N, Mocanu MM, Yellon DM (2013). Dipeptidyl peptidase-4 inhibitors and GLP-1 reduce myocardial infarct size in a glucose-dependent manner. Cardiovasc Diabetol.

[28] Kyhl K, Lønborg J, Hartmann B, Kissow H, Poulsen SS, Ali HE, Kjær A, Dela F, Engstrøm T, Treiman M (2017). Lack of effect of prolonged treatment with liraglutide on cardiac remodeling in rats after acute myocardial infarction. Peptides.

[29] Astrup A, Carraro R, Finer N, Harper A, Kunesova M, Lean ME, Niskanen L, Rasmussen MF, Rissanen A, Rössner S, Savolainen MJ, Van Gaal L, NN8022-1807 Investigators (2012). Safety, tolerability and sustained weight loss over 2 years with the once-daily human GLP-1 analog, liraglutide. Int J Obes.

[30] Wadden TA, Hollander P, Klein S, Niswender K, Woo V, Hale PM, Aronne L (2013). Weight maintenance and additional weight loss with liraglutide after low-calorie-diet-induced weight loss: the SCALE maintenance randomized study. Int J Obes.

[31] Pi-Sunyer X, Astrup A, Fujioka K, Greenway F, Halpern A, Krempf M, Lau DC, le Roux CW, Violante Ortiz R, Jensen CB, Wilding JP, SCALE Obesity and Prediabetes NN8022-1839 Study Group (2015). A randomized, controlled trial of 3.0 mg of liraglutide in weight management. N Engl J Med.

[32] Nauck MA, Kleine N, Orskov C, Holst JJ, Willms B, Creutzfeldt W (1993). Normalization of fasting hyperglycaemia by exogenous glucagon-like peptide 1 (7–36 amide) in type 2 (non-insulin-dependent) diabetic patients. Diabetologia.

[33] Russo GT, Labate AM, Giandalia A, Romeo EL, Villari P, Alibrandi A, Perdichizzi G, Cucinotta D (2015). Twelve-month treatment with Liraglutide ameliorates Visceral Adiposity Index and common cardiovascular risk factors in type 2 diabetes outpatients. J Endocrinol Invest.

[34] Drucker DJ (2018). The ascending GLP-1 road from clinical safety to reduction of cardiovascular complications. Diabetes.

[35] Hue L, Taegtmeyer H (2009). The Randle cycle revisited: a new head for an old hat. Am J Physiol Endocrinol Metab.

[36] Lorenz M, Lawson F, Owens D, Raccah D, Roy-Duval C, Lehmann A, Perfetti R, Blonde L (2017). Differential effects of glucagon-like peptide-1 receptor agonists on heart rate. Cardiovasc Diabetol.

[37] Zhang LH, Pang XF, Bai F, Wang NP, Shah AI, McKallip RJ, Li XW, Wang X, Zhao ZQ (2015). Preservation of glucagon-like peptide-1 level attenuates angiotensin II-induced tissue fibrosis by altering AT1/AT 2 receptor expression and angiotensin-converting enzyme 2 activity in rat heart. Cardiovasc Drugs Ther.

[38] Zhao T, Chen H, Xu F, Wang J, Liu Y, Xing X, Guo L, Zhang M, Lu Q (2019). Liraglutide alleviates cardiac fibrosis through inhibiting P4 hα-1 expression in STZ-induced diabetic cardiomyopathy. Acta Biochim Biophys Sin.

[39] Aoyama M, Kawase H, Bando YK, Monji A, Murohara T (2016). Dipeptidyl peptidase 4 inhibition alleviates shortage of circulating glucagon-like peptide-1 in heart failure and mitigates myocardial remodeling and apoptosis via the exchange protein directly activated by cyclic AMP 1/Ras-related protein 1 axis. Circ Heart Fail.

[40] Lehrke M, Marx N (2017). Diabetes mellitus and heart failure. Am J Cardiol.

[41] Weir DL, McAlister FA, Senthilselvan A, Minhas-Sandhu JK, Eurich DT (2014). Sitagliptin use in patients with diabetes and heart failure: a population-based retrospective cohort study. JACC Heart Fail.

[42] Sposito AC, Berwanger O, de Carvalho LSF, Saraiva JFK (2018). GLP-1RAs in type 2 diabetes: mechanisms that underlie cardiovascular effects and overview of cardiovascular outcome data. Cardiovasc Diabetol.

[43] Tanaka A, Node K (2018). Clinical application of glucagon-like peptide-1 receptor agonists in cardiovascular disease: lessons from recent clinical cardiovascular outcomes trials. Cardiovasc Diabetol.

[44] Dawwas GK, Smith SM, Park H (2018). Risk of heart failure hospitalization among users of dipeptidyl peptidase-4 inhibitors compared to glucagon-like peptide-1 receptor agonists. Cardiovasc Diabetol.

[45] Koyani CN (2017). Dipeptidyl peptidase-4 independent cardiac dysfunction links saxagliptin to heart failure. Biochem Pharmacol.

[46] Mulvihill EE (2016). Inhibition of dipeptidyl peptidase-4 impairs ventricular function and promotes cardiac fibrosis in high fat-fed diabetic mice. Diabetes.

[47] Gardiner SM, March JE, Kemp PA, Bennett T (2006). Mesenteric vasoconstriction and hindquarters vasodilatation accompany the pressor actions of exendin-4 in conscious rats. J Pharmacol Exp Ther.

